# Estimating the Mass of Food Components Necessary for the Utilization of Free Radical Particles in the Human Body

**DOI:** 10.3390/ijerph192315574

**Published:** 2022-11-23

**Authors:** Victor Gorbachev, Evgeny Nikulchev, Alexander N. Kosenkov, Andrey Sokolov, Igor Zavalishin, Igor Nikitin

**Affiliations:** 1 Department of Biotechnology of Food Products from Plant and Animal Raw Materials, K.G. Razumovsky Moscow State University of Technologies and Management (The First Cossack University), 73 Zemlyanoy Val, 109004 Moscow, Russia; 2 Department of Digital Data Processing Technologies, MIREA—Russian Technological University, 119454 Moscow, Russia; 3 Department of Hospital Surgery, Sechenov First Moscow State Medical University, 119435 Moscow, Russia; 4 Mental-Health Clinic No. 1 Named after N.A. Alexeev, 117152 Moscow, Russia; 5 Department of Automated Control Systems, K.G. Razumovsky Moscow State University of Technologies and Management (The First Cossack University), 73 Zemlyanoy Val, 109004 Moscow, Russia

**Keywords:** antioxidants, vitamins, act of breathing, free radicals, oxygen, xenobiotics, smoking, alcohol, paraquat, nutritional therapy, foods, NAC, vitamin C

## Abstract

The article proposes an algorithm for an approximate assessment of the molar volume of free radicals generated in the human body per day. It takes into account the act of breathing, physical activity, food consumption, the influence of unfavorable environmental conditions, exposure to xenobiotics, as well as bad habits (alcohol and tobacco smoking). A calculation of the required set of the most commonly used food products for the disposal of free radicals was made. The calculation is a structure of four blocks with the possibility of adding optional data from human population genetic studies, environmental conditions, etc. In the proposed algorithm, the results of antiradical activity (ARA) of food products are used as input, including the results of predicting antiradical activity using artificial neural networks (ANN), which we published earlier. Based on the accepted values of one equivalent (in terms of the activity of 1 μmol of ascorbic acid), it was shown (for our data) that for the utilization of all free radicals produced in the human body per day, it will take an average of ≈260 to ≈540 g of food components in terms of dry mass (including proteins, fats, carbohydrates, etc.). At the same time, for the utilization of consumed xenobiotics, from 220 mg (in terms of vitamin C) to 260 mg (in terms of acetylcysteine -NAC) of additional plastic components or 11.5–13.0 g of essential amino acids will be required, which must be taken into account when calculating diets. This approach will be useful in the development of new functional foods, as well as in assessing the possible impact of diets on human health. Another applied point of this study is related to the possibility of using these data for better detailing and selection of food products for people working in conditions of increased radiation (in space conditions), in contact with harmful substances (chemical synthesis and production), for people practicing increased physical activity (bodybuilding and sports), and for the purposes of medical nutritional therapy.

## 1. Introduction

One of the main sources of free radicals in the human body is the act of breathing (including cellular) [1,2,3]. Oxygen consumed during respiration manifests itself in two ways: on the one hand, it is necessary for the processes of oxidation and the production of macroergic molecules (for example, Adenosine triphosphate (ATP)), and on the other hand, it is a source of formation of “reactive oxygen species” (hereinafter ROS) [2,4]. According to published data, approximately 0.15% of the gas consumed by cells in vivo is in the ROS state [4,5].

Around the middle of the 1950s, the theory of free radical aging and the formation of a number of human pathological conditions associated with the generation of free radicals was formulated [6].

Since the publication of this article, additional studies have been carried out and data have been accumulated on the study of free radical pathogenesis of a number of diseases, namely diseases of the cardiovascular system, atherosclerosis, hypertension, Alzheimer’s disease, chronic connective tissue diseases, etc. [7].

Among other things, studies have been devoted to understanding the chemical processes of the formation of summary radicals associated with bad habits of people, such as smoking tobacco and drinking alcohol, the presence of xenobiotics in food raw materials and food products, and other adverse environmental and ecological factors, for example, as shown in [6,7,8,9,10], and many others, which aggravated the course of the disease in people.

With the accumulation of data, it became clear that free radical chain reactions, including those in lipid fractions, can be stopped (elimination of the reaction course).

For this reason, in the 1960s–1970s, research aimed at searching for chemical components (substances) capable of stopping free radical chain reactions, which were called “radical traps”, became relevant [1,3,4,11]. A little later, approximately in the 1980s–1990s, publications in which enzymatic systems were studied and implemented in the antiradical defense strategy by the cells of the human body became widespread [9,10].

Implemented by man, the mechanisms of protection against free radicals involve both enzymatic activity and the action of low-molecular-weight substances with antiradical properties. Among the latter, it was found that a certain proportion of them enters the human body with food [11,12].

Such substances include food components such as vitamins (A, C, B, E, etc.), polyphenolic compounds (flavonoids, anthocyanins, etc.), amino acids, some components of lipids and polysaccharides, etc. [7,11].

Due to the decline of the environmental situation in a number of regions of the world, the relevance and the need for people to develop diets with increased protective properties have increased (food systems that contain antioxidants as additional biologically active substances). For this reason, there has been an increase in the amount of research aimed at evaluating and searching for antiradical substances in food systems, which was facilitated by the development of appropriate methods [4,7,11].

To date, methods have been developed for assessing the antiradical activity (ARA) of various nutrients (for example, using photocolorimetric approaches) [12,13,14,15]. Thus, the potential of each of the listed groups of nutrients (vitamins, polyphenols, etc., see above) can be predicted with a high degree of accuracy [16,17].

Since the stoichiometric ratios of the interaction of radicals and antiradicals are also known, it is possible to develop an algorithm that assumes an approximate estimate of the mass of radicals produced by the human body and the required average volume of plastic components of food systems that can interact with the produced radicals and neutralize them [1,3,9,10,11,15].

Thus, the assumption of the possibility of developing diets for wide public consumption, in order to reduce the propathogenic effects of free radicals on the human body, is relevant [15]. However, for this it was necessary to obtain multiple statistical data. For this reason, earlier, we were forced to divide this study into research blocks: (1) to make primary estimates of ARA, or food consumed. This was done using photocolorimetry of standardized DPPH free radical solutions and published by us previously in the literature [15]. In the above study, we identified 11 food groups (fruits and vegetables, cereals, legumes, dairy products, meat products, fish and seafood, eggs, confectionery, carbonated drinks, tea and coffee, biologically active substances from wild plants, etc.) from 15 countries located in different parts of the world: Great Britain, the USA, China, Belgium, Paraguay, India, Ecuador, Mexico, Poland, Moldova, Egypt, the Netherlands, Belarus, Thailand, Denmark, and Russia. In total, according to Google statistics, these countries account for just over 3.7 billion people, i.e., 48% of the world’s population, which, in principle, allows us to use these data when predicting the properties of food products from other countries that we have not studied.

After collecting laboratory data, the next stage of research was the prediction of ARA based on the chemical composition of foods using neural networks (ANN).

The study included popular complex and simple dishes from Asian, European, and American cuisines with a total of more than 1300 items [16]. In the work, we used data on the content of proteins, fats, carbohydrates, vitamins, and mineral components (for example, selenium) in foodstuffs, etc., in a total 60 items of substances. In the course of this study, the uneven contribution of various components of food systems to the final values of ARA (which is important in food engineering) was shown; these data were also published earlier [15,16]. The obtained estimates were also necessary for understanding the average ARA value of food consumed by an average person from different countries regardless of his culinary preferences (average values).

However, in order to create new types of functional nutrition, it is necessary to make at least approximate estimates of the amount of free radicals produced in the human body. Thus, the logical conclusion of these studies will be the creation of an algorithm for an approximate assessment of the generated particles and food components that can utilize them. The creation of it is the goal of this study.

The integrated approach implemented by us, which involves both laboratory studies to assess the ARA of food and the use of ANN, with the subsequent creation of a mathematical algorithm for estimating the volume of free radicals and food components necessary for their utilization, was not previously encountered in the literature.

We note right away that enzymes are also an important anti-radical barrier in the human body. However, their activity depends on the presence of certain genetic mutations in the human genome, and additional population studies are needed to assess enzymatic activity. Nevertheless, we assumed the possibility of using such data in future works (this block is not taken into account in the present calculations).

The study is organized as follows: Section 2 provides materials and methods; Section 2.1 provides description of the research units; Section 2.2 contains data for calculations for the act of breathing; Section 2.3 has input data for calculating the generation of free radicals in smokers; Section 2.4 has input data for calculating volumes of generation of radicals in humans when consuming xenobiotics and alcohol; Section 3 describes the results and discussions; Section 3.2 provides calculation of the mass of plastic substances from food, which is necessary for the utilization of xenobiotics; and Section 4 includes some explanation and the conclusion.

## 2. Materials and Methods

The data published in the literature (for example, [1,2]), including those previously obtained by us [13,15,16], served as materials for the development of the study.

As is known, numerous forms of antioxidant protection are implemented in the human body [5,11,12]. Among them there are both enzymatic and those associated with the interaction of particles with a small molecular weight (for example, lipids and reactive oxygen species, ROS) [2,6,11].

In view of the fact that the activity of enzymes is genetically determined, it is not possible to make an accurate assessment of their antioxidant activity bypassing the stage of population genetic studies [3,4,11].

For this reason, we coarsened the input assumptions for this calculations. Estimates are made in it as if the interaction inside the human body took place only between free radicals and a pool of antioxidants or other food components, bypassing the enzymatic activity of cells. Such an approach, despite its roughness, is similar to studies carried out earlier in vitro [15], where the chemical composition and their molar ratios were primarily important.

The input theoretical data for this study is formed of, first of all, physical constants, the composition of food products as well as data on the antiradical activity of food products predicted by neural networks based on the composition of food components of the international food database (FNDDS) [16]. The main calculation and statistical analysis of the data (for example, 95% confidence interval) was carried out using Excel 2010.

### 2.1. Description of the Research Units

**Figure 1:**
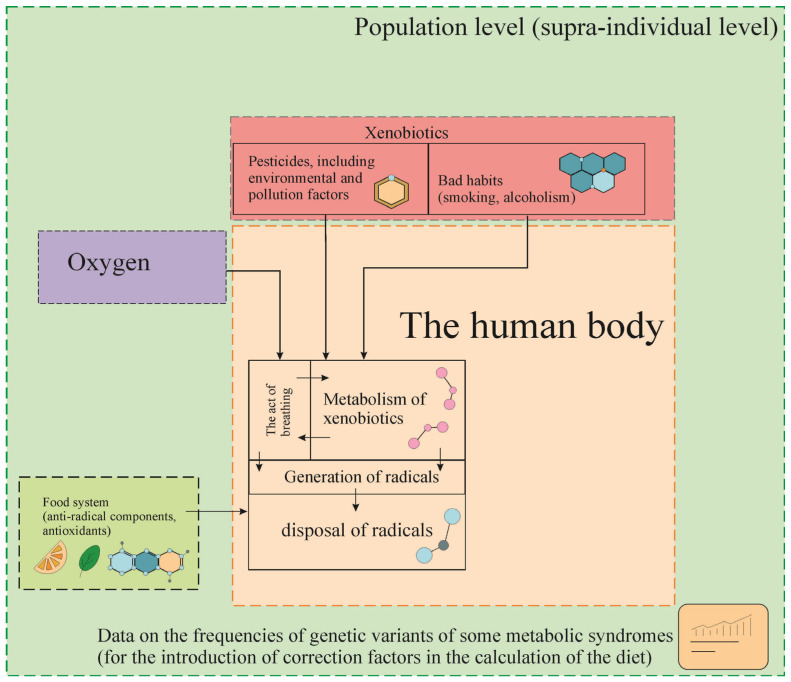
A diagram illustrating the stages of research to calculate the volume of production of free radicals and food components capable of reacting with them.

Figure 1 schematically shows the main ways of generating free radicals and methods of their neutralization.

The purpose of the study is to identify the dependence of the occurrence of radicals and to find compensatory nutritional components that ensure the neutralization of radicals.

Let *P* be the volume of incoming radicals; then, it is required to find a compensation that ensures the preservation of a constant value of radicals in the body (according to the approximate equality below):

(1) $$P\left(m,\;o\right)-\sum_{i=1}^{n}X_{i}\left(m,\;o\right)\;\approx const$$

where *P* is a function that depends on many parameters, and given its use in the present work for the formation of a specialized diet, we will consider it dependent on the mass *m* of the oxygen consumed, and the parameter *o* represent specialized characteristics (age, gender, smoking, etc.); the sum of compensatory *X_i_* functions is the value necessary for the neutralization of radicals based on the *i*-th nutritional components.

Since *o* determines not only the value of the functions *P* and *X_i_* but also *m*, we will evaluate the value of the functions by fixing (sex, age, smoking, sports) *o*. This will allow for each chosen *o* to obtain estimates of *P* in the form of numerical values.

In other words, for

(2) $$\forall o:\;\exists P\left(m,o\right)=r\in \mathbb{R}$$

Estimation of the numerical value of the moles (mass) of radicals for a particular case will allow you to choose food components as the sum of compensatory neutralizing elements (taking into account the anti-radical capacity of food products). The problem of choosing *X_i_* does not have an unambiguous solution, but any combination that adds up to the found value of *r* (radicals) will make it possible to ensure the neutralization of excess radicals that have entered the body.

Thus, to solve the problem:

(1) It is necessary to choose a set of essential parameters *o* that have a significant impact on the estimate of the oxygen mass;
(2) To develop a scheme for calculating the values *r* = *P* (*m*, *o*) for given *o* values;
(3) Choose a set of *X_i_* nutrients that significantly affect the neutralization of radicals;
(4) Offer a set of *X_i_*, the sum of which provides compensation *r*.

To obtain estimates of the volume of radicals *P*, we will consider the following cases (men/women, sports/sedentary lifestyle, smoking, and consumption of xenobiotics and alcohol). Below are estimates of volumes for the indicated parameters.

### 2.2. Input Data for Calculations for the Act of Breathing

As noted above, approximately 0.15% of the consumed oxygen passes from the triplet form (not excited) to the form of free radicals due to metabolic processes, which contributes to the initiation of a chain of radical reactions [4,5].

Physiological studies have shown the existence of a relationship between a person’s weight, his physiological activity, gender, age, and the amount of oxygen consumed (O_2_). These data are presented in Table 1. For the purposes of this study, we have chosen the average age groups of 35–44 years as one of the most socially active age periods [18].

Knowing the density of oxygen, taken according to table values, which under normal conditions are 1.42897 g/L, it is possible to calculate the mass of oxygen consumed per day and, as a result, that part of it that goes into the state of free radicals. As an example, let us take the average weight of representatives of the adult population of Russia. According to recent studies, it is 82.2 kg for men and 72.6 kg for women [19]. These values are comparable for a number of countries both in Europe, Oceania, and North America [20,21,22,23,24].

### 2.3. Input Data for Calculating the Generation of Radicals in Smokers

Previously, a relationship was shown between the probability of occurrence of cardiovascular diseases in a person and his propensity to smoke [8]. This kind of dependence is dictated by the direct effect of free radicals formed during the thermal decomposition of tobacco and inhalation of tobacco smoke. Input data for calculations are presented in Table 2.

As can be seen from Table 2, the methylidine radical (HC^3e−^), with three unpaired electrons, has the greatest mol rate (almost ½). Knowing the approximate value of one smoker’s puff volume—on average 45–55 (up to 90 mL) of smoke [8]—and the average number of puffs per cigarette—12–14 [25]—let us recalculate the values of the chemical composition of tobacco smoke per one smoked product (cigarette). Thus, for one cigarette in 95% CI, there are from 540 to 770 mL (up to 1260 mL) of tobacco smoke or 1.86–2.65 mg (up to 4.34 mg) of xenobiotics, corresponding to the values presented in Table 2. Moreover, 67% of their weight falls on three valence methylidine radicals, which increases their reactive potential by 1.89 times on average. Let us make a weighted average recalculation for each position, which in total will give 1.13 × 10^−4^–1.61 × 10^−4^ mol (up to 2.63 × 10^−4^ mol) of xenobiotics when smoking one cigarette.

### 2.4. Input Data for Calculating Volumes of Generation of Radicals in Humans When Consuming Xenobiotics and Alcohol

In the production of agricultural raw materials, a huge number (more than 1000) of various types of pesticides is currently used. In addition to these means, modern man is faced with more than 60 thousand different chemical components [9,26,27].

Studies on the assessment of pesticide and aflatoxin residues in agricultural raw materials are carried out annually as monitoring, and data on toxicology and the content of these substances in food products are published [26,27,28,29]. There is evidence of an annual increase in the content of these substances, so the data for 2021 will be relevant.

According to the FAO report on the content of pesticide residues in food raw materials for 2021, it was shown that their amount can vary for different types of plant raw materials. For example, leaf basil, spinach, parsley, mustard, leaf lettuce, coriander, marjoram, and cumin contain 47.18 mg of xenobiotics/kg of product (hereinafter, in total for some groups of substances). The residual content in apples and pears, pumpkins, cucumbers, and marrows is 0.09 mg/kg of raw material. In cereals and products of their processing, it is 0.04 mg/kg. Bananas contain 0.19 mg/kg of raw material. In berry crops, for example, blackberries and raspberries, there is 0.26 mg/kg of raw material. In citrus fruits, it is 0.73 mg/kg, while a number of products of their processing, for example, essential oils, can contain several orders of magnitude more, up to 150 mg/kg of the product [26,27].

In addition to pesticides, we will take into account such components of food systems as nitrates, which can also contribute to malignancy of gastrointestinal cells [30]. According to recent studies, the average values for the content of this component in food raw materials, for example, for the Siberian region, is 262 mg/kg of raw materials in total for different groups of vegetables and fruits. In this publication, we provide calculated values for nitrate intake of 170 mg/day/1 person [30], which is confirmed with values in other published studies [31,32]. At the same time, it is known that, for example, boiling can reduce, on average by ≈12.2%, the values of the nitrate concentration in the processed raw materials, while frying, on the contrary, increases the proportion of residual nitrates [33].

Obviously, it is not possible to make an accurate assessment of the effect of all these groups of substances on the human body, while it is possible to use reference substances, for example, such as paraquat [9,11,34,35]. This type of pesticide has long been one of the most widely used in the world [34]. According to the mechanism of chemical action, it belongs to the so-called “uncouplers of respiration” [9,35].

The ingress of this substance into the human body leads to the constant generation of free radicals until it is utilized. One of the main substances, whose volume drops sharply in case of pesticide poisoning, is glutathione or its precursors such as cysteine derivatives [9,36]. All of the above allows us to apply research on the mechanism of paraquat poisoning for analysis in our calculations.

For example, studies on the survival of laboratory animals have shown a positive effect of acetylcysteine (NAC) on the increase in resistance during the generation of free radicals [36]. It was found that in case of poisoning with paraquat (70 mg/kg of body weight) for every 0.4 mL of blood, free radicals are generated in the amount of 5.2 to 10 μmol for every 4 h of intoxication. For a day, this gives the production of radicals in the amount of 0.4–0.78 mol and 0.3–0.59 mol at the given parameters of poisoning for a man (5200 mL of blood) and for a woman (3900 mL of blood), respectively. This makes it possible to stoichiometrically calculate the number of equivalents of antiradical food components per 1 mg of a pesticide or any other (roughly) xenobiotic that generates free radicals and has the potential to enter the human body.

It should also be taken into account that some of cigarette smoke and alcohol components or xenobiotics will generate additional free radical particles until the time of their complete utilization (excretion or termination in tissues); thus, when toxic agents enter the human body, not only their dose is important but also the excretion period (half-life), which, ultimately, is not possible to calculate in an accurate way. All this leads to an underestimation of the obtained values.

When taking alcohol (including poisoning with ethyl alcohol), the mechanism of generating free radicals does not fundamentally differ from poisoning by other xenobiotics. That is why we will make calculations for ethyl alcohol in the same section. Previously, it was shown in literature that from 25 to 50% (37.5% on average) of ethanol molecules can be oxidized to acetaldehyde via the monovalent hydroxyethyl radical [10], which makes it possible to estimate the equimolar masses of antiradical substances necessary for the utilization of reactive particles generated by the intake of alcohol-containing food.

## 3. Results and Discussion

A summary estimate of the values of *P (m, o*) calculating for the proposed algorithm is given in Table 3.

Taking into account the volume of oxygen consumption by a person (Table 1) and the density of this gas (1.42897 g/L), it was found that a person consumes on average from 4.4 to 7.7 kg of oxygen per day (Table 3). Taking into account the fraction (0.0015) of the transition of oxygen into free radicals and the molar mass of 16 g/mol for oxygen, we calculate the quantity of mol of radicals according to the equation:

(3) $$n_{\mathrm{rb}}={\;\mathrm{VO}}_{2}\times {\rho O}_{2}\times m_{\mathrm{hb}}\times {\%}_{\mathrm{rad}}$$

where n_rb_ is the number of generated radicals in the human body in moles for a certain constant period of time during breathing, VO_2_ is the volume of oxygen consumed by a person over a certain constant period of time per/kg of weight, ΡO_2_ is the air density in g/ml, %_rad_ is the conversion coefficient (share) of oxygen converted into free radicals during the act of breathing = 0.0015, and m_hb_ is the mass of the human body/kg.

The mass of radicals is calculated according to the equation:

(4) $$m_{r}=\left(n_{\mathrm{rb}}\times m_{o})+(n_{\mathrm{rx}}\times {\;m}_{\mathrm{xb}}\right)+\cdots +(n_{x}\times m_{x})$$

where m_r_ is the mass of radicals in total (in grams); n_rb_ is the number of radicals generated in the human body in moles during breathing; m_o_ is the molar mass of oxygen; n_rxb_ is the number of radicals generated in the human body when xenobiotics enter it in moles; m_xb_ is the molar mass xenobiotics, n_x_; m_x_ is the corresponding number of moles and the mass of other radical particles not taken into account by us but possible to be taken into account when adding the appropriate blocks in research (for example, population genetic data, data on the generation of free radicals during infection with viral diseases, etc.).

During the mentioned above time period, from 0.42 to 0.72 mol of radicals are generated in the human body. Taking into account the previously accepted value of 1 equivalent of antioxidants equal to 1 μmol of ascorbic acid [13,14,37], the obtained values correspond to from ≈ 2.3 to ≈ 4.1 thousand equivalents necessary for the utilization of generated free radical particles. It follows that it is possible to calculate the mass of the desired food component or the sum of the components (for a whole food product or for a diet) according to the following equation:

(5) $$M_{\mathrm{FC}}=\frac{n_{\mathrm{srb}}}{\frac{{\mathrm{Eq}}_{c}}{{\mathrm{Eq}}_{f}}}$$

where M_FC_—the mass of food components necessary for the utilization of free radicals in human body; n_srb_—the number of generated radicals in the human body in moles for a certain constant period of time in total from all sources; Eq_c_—the amount of anti-radicals substances in moles (in our study 1 equivalent = 1 μmol of ascorbic acid) for calculating the reduction capacity; Eq_f_—the anti-radical capacity of a food product or food component, measured in equivalents (Eq_c_); see articles for more [14,15,16].

Similar calculations are made in accordance with the input data for each of the blocks. Some difficulties in these calculations lie in the fact that the pool of antiradicals for evaluating xenobiotics during smoking, in fact, will refer only to water-soluble substances such as glutathione, cysteine, vitamin C, or polyphenolic compounds, and it is not entirely correct to extend the general physiological values to other groups; however, hydroxyethyl radicals have a known ability to interact with the lipid fraction [9,10]. In other words, at present, we do not have full-fledged empirical data to understand the proportion in which hydrophilic and hydrophobic antioxidants and free radicals interact with each other. However, these data can be included in the algorithm proposed by us in the future with the accumulation of new information. Note also that if we are talking about sulfur-containing amino acids (cysteine and its derivatives), then the values in Table 3 should be doubled (since they are monovalent).

Let us calculate food components *X_i_* and the mass of food products (averaged) necessary for the utilization of free radicals generated in the human body as if remaining components did not participate in the reactions of free radical termination.

For the analysis, we take generalized data on food products. Previously, using artificial neural networks (ANNs), we predicted the antiradical potential for 1315 foods from 10 food groups based on the FNDDS database [16,38]. The average value for all food products was ≈25.3 (and ranged from 23.0–27.6 in 95% CI) equivalents (per ascorbic acid) per gram of dry product. Recalculations were made taking into account an average moisture content of 66.9% and a correction factor for residual antiradical activity of 34.5% (unpublished data). The obtained values are presented in Table 4.

### 3.1. Evaluation of the Impact of Xenobiotics on Final Values

As indicated above, a number of xenobiotic substances enter the body of an average consumer due to objective reasons of agricultural production. A number of pesticides have a long half-life, forming chronic poisoning. However, for the purposes of this study, we will roughly assume the half-life period taken for the initial stage of paraquat poisoning to be 12 h [34,36].

Let us estimate the average content of pesticides, taking into account the above values. It ranges from 0.26 to 8.08 mg/kg of products. We also take into account the mass of incoming nitrates in 170 mg/kg of raw materials [30], but we will take a lower value as if all food products were cooked by boiling (−12.2% nitrates) [33]. Taking into account the fact that vegetables and fruits account for approximately 25% of the total diet (we summarize pesticides separately, since they can also be found in animal products), we calculate the final value, which ranges from 37.6 to 45.4 mg/kg of food consumed. According to the consumption standards adopted in Russia (which are close to the U.S.’s and Codex Alimentarius values), one person accounts for approximately from 1540 to 3160 g of finished products [39,40], which gives from 88 to 107 mg of xenobiotics (on average 97.5 mg) per day. The average volume of food consumed is taken as 2350 g per person.

Consumed substances will increase the lower threshold of generated radicals by an average of 52.2 ARA equivalents. We will carry out a similar calculation for the case of a person consuming 50 g of ethyl alcohol and smoking 1 pack of cigarettes per day (20 products), which, together with xenobiotics, will give an average of 1222.7 equivalents more and require an appropriate amount of antiradical substances.

### 3.2. Calculation of the Mass of Plastic Substances Necessary for the Utilization of Xenobiotics

The calculation is made on the base of molar ratios to the mass of consumed nutrients (primarily NAC and vitamins C and B group) since it was previously shown that they exhibit significant activity in food systems [11,15,16]. Protein-containing components were taken for calculation due to their nutritional value. The data are shown in Table 5.

This table contains primarily data on amino acids vitamins added to it for comparison (water-soluble). This is dictated by the fact that the main molar fraction of antiradical components in the human body is glutathione [41]. If we take into account, according to these data, the rate of its synthesis of ≈0.229 g of this antiradical per day per liter of blood and the daily requirement of an adult in 1.32 g of cysteine [42], then the molar ratios of all vitamins consumed per day [39] are approximately from 3.6 to 4.8 times less than cysteine, which is directed to the utilization of free radicals in the form of glutathione.

According to the data presented above (Table 5), actions aimed at fortifying foodstuffs with vitamins and protein (also as a part of the diet for fitness people) are reasonable.

Previously, we suggested that ARA depends on several factors, for example, the molecular weight and hydrophobicity/hydrophilicity of the food component and its ionicity [15]. As we can see, low-molecular-weight structures are more active in relation to free radicals, which are correlated with the calculated mass of food components. Thus, for example, according to Table 5, substances with a low molecular weight, such as NAC (as a derivative of cysteine), free amino acids, ascorbic acid, complex vitamin preparations, etc., require much less (up to 53 times even when comparing ascorbic acid and free amino acids). This is largely due to the higher ionic strength of ascorbic acid relative to essential amino acids Table 5, which are most often more hydrophobic.

As we have shown earlier, the ARA of protein-containing food products increases in the following order: native food proteins < protein-enriched hydrolysates < a mixture of molecular amino acids (with rare exceptions, for example, in relation to methionine, which, according to our data, showed an extremely weak ARA despite the presence of sulfur in its molecule, which is very different from substances such as acetylcysteine or glutathione) [15].

Adding these substances to the diet could be a way to increase the amount of “anti-radical traps” in food. However, the same free amino acids in high concentrations can cause side effects since it is known that they have a “narrow therapeutic index”—this applies primarily to sulfur-containing amino acids such as methionine and cysteine [43]. All this leads to the need to use low molecular weight individualized substances with caution when formulating diets. There is another conclusion, namely the low expediency (both from the point of view of pharmacology and from the point of view of human physiology) of the use of monomolecular amino acids for the complete replacement of food raw materials. At the same time, some derivatives (for example, acetylcysteine) can be quite successfully used to improve the AR properties of food products [15].

## 4. Conclusions

This algorithm demonstrates the potential for practical use in the development of individual diets or for medical dietary therapy (MHT) to reduce the severity of pathogenic manifestations of diseases, for example, in certain types of cardiovascular diseases.

Thus, resent studies showed that the likelihood of certain types of heart pathologies and atherosclerosis can be reduced by the consumption of antioxidants, such as flavan derivatives, etc. [44,45]. For this reason, the most promising for the development of rations and products are polyphenol compounds: quercetin, muracetin (a number of other flavan derivatives), vitamin preparations (C, B), extracts (galenic preparations) from wild plants, and derivatives of sulfur-containing amino acids and their metabolites (including taurine, acetylcysteine), including in their mixtures [11,17,45,46,47]. Previously, we have shown a relationship between the ARA of food products and such components as dietary fiber, folic acid, β-cryptoxanthin, etc. [16]. This shows the possibility of developing a variety of diets using new non-trivial types of substances with ARA, and their search is one of the tasks in this area of research.

We believe that in addition to the development of innovative products from various commodity groups, it is necessary to correct diets for those consumers who have an unbalanced diet, bad habits, or live in an unfavorable environmental conditions. The following product groups show great prospects for development on their basis: carbonated sweet drinks, bakery products, freeze-dried cereals, and dairy products etc., provided that they are supplemented with appropriate substances with increased antiradical activity.

However, we do not exclude the possibility of using this research in the development of diets for patients with free radical lung pathologies, etc. We believe that the estimations we propose can be applied for professional nutrition purposes, for example, for people working in areas with high radiation, in space, with increased levels of physical activity (for example, for bodybuilders), etc.

The calculation we have made currently does not sufficiently detail secondary radicals, and we do not yet understand how radicals can be included, for example, generated during certain types of diseases, for example, when infected with COVIID-type viral infections; at the least, this requires additional research.

Undoubtedly, this assessment approach can be expanded, and data on human population genetic studies can be added to it as a separate block. In addition to the above, blocks for changes in nutritional value during food storage, data on the chemical composition of products, and detailed data on the generation of free radicals when various types of xenobiotics enter the human body can apparently be added to the calculations as correction factors.

In conclusion, we note in addition that the restrictions on the use of food components from Table 5 in some cases include the use of large dosages of sulfur-containing amino acids. This is especially true if a person has a congenital pathology of the metabolism of sulfur-containing amino acids (for example, cystinuria, etc.), which requires consultation with the attending physician.

## Figures and Tables

**Table 1:** The volume of oxygen consumed (VO_2_ in mL/kg/min) depending on gender and physical activity, given for one age group (35–44 years), according to literature data [18].

No.	Gender	Physical Activity	VO_2_ mL/kg/min (95% CI)
1	Male	High (athletic lifestyle)	44.8–46.0
2	Male	Low (sedentary lifestyle)	38.8–41.0
3	Female	High (athletic lifestyle)	34.1–35.7
4	Female	Low (sedentary lifestyle)	29.0–31.0

**Table 2:** Average values of the chemical composition of tobacco smoke, in terms of M^3^ and mol of radicals, respectively [8].

No.	Substance ^1^ (Molar Mass)	mg/M^3^ Mean	95% CI (Min)	95% CI (Max)	mol ^2^	In % of All Moles
1	CO (28)	636.3	532.6	740.1	0.02273	20.71%
2	CO_2_ (44)	1263.3	991.8	1534.9	0.02871	26.16%
3	NO (30)	18.7	16.0	21.3	0.00062	0.57%
4	NO_2_ (46)	1.1	0.7	1.5	0.00002	0.02%
5	NO_x_ (30_x_)	19.7	17.8	21.6	0.00066	0.60%
6	HC^3e−^ (13)	645.0	595.2	694.8	0.04962	45.20%
7	Mixture of components ^3^	860.5	754.8	963.4	0.0074	6.75%

Note: ^1^ aerosols; ^2^ average value in mol; ^3^ ≈ 90% of them are acetone, nicotine, methylfuran, toluene, and glycerol or ≈0.333 mg of organic impurities per 1 mg of particulate smoke.

**Table 3:** Summary table of calculating *P (m, o)* and ARA equivalents for the proposed algorithm.

Calculation of Physiological Equivalents				
No.	Data from the Table 1	Mass of Oxygen Consumed per Day, in Grams	Mole Radicals	Calculation of ARA Equivalents
1	Male (athletic lifestyle)	7679.2	0.72	4087.67
2	Male (sedentary lifestyle)	6748.9	0.63	3592.46
3	Female (athletic lifestyle)	5213.7	0.49	2775.30
4	Female (sedentary lifestyle)	4481.7	0.42	2385.64
**Calculation of equivalents when smoking**				
5	For 1 cigarette (CI 95% Min)		1.13 × 10^−4^	0.64
6	For 1 cigarette (CI 95% Max)		1.61 × 10^−4^	0.91
7	For 1 cigarette at maximum puff V^3^		2.63 × 10^−4^	1.49
**Calculation of equivalents when taking alcohol**				
8	When taking 1 g of 95.6% ethyl alcohol (with an oxidation fraction of 0.375)		0.0078	22.1
**Calculation of equivalents when consuming xenobiotics (per 1 mg)**				
9	For a man (CI 95%)		1.2–2.3 × 10^−5^	0.4–0.76
10	For a woman (CI 95%)		0.9–1.2 × 10^−5^	0.33–0.65

**Table 4:** Calculated values of the mass of food products and components with ARA required for the utilization of generated free radicals.

No.	Physical Activity	Mass of Food Components, in Grams	
		Mean	95% CI (Min–Max)
1	Male (athletic lifestyle)	488.1	447.4–536.9
2	Male (sedentary lifestyle)	429.0	393.2–471.9
3	Female (athletic lifestyle)	331.4	303.8–364.5
4	Female (sedentary lifestyle)	284.9	261.1–313.4
**The mass of food components necessary for the disposal of ethyl alcohol oxidation products, in grams ^!^**			
5	Mass of vitamin C (per 1 mL 95.6%)	0.0038 0.011	
6	Mass of acetylcysteine (NAC) (per 1 mL 95.6%)	0.0072 0.021	
**The mass of food components necessary for the utilization of xenobiotics and smoking products** *			
5	Mass of vitamin C (xenobiotics per 1 mg)	88 μg 255 μg *	55–126 μg 159–365 μg *
6	Mass of vitamin C (smoking products for 1 cigarette)	136 μg 394 μg *	113–161 μg 385–466 μg *
7	Mass of acetylcysteine (NAC) (xenobiotics per 1 mg)	190 μg 550 μg *	120–272 μg 346–788 μg *
8	Mass of acetylcysteine (NAC) (smoking products for 1 cigarette)	252 μg 730 μg *	210–298 μg 608–862 μg *

Note: * taking into account the correction factor of the residual ARA; ^!^ calculated values according to average literary values [10].

**Table 5:** Masses of main low-molecular-weight substances from food products necessary for the utilization of xenobiotics, without taking into account the possible ARA of other food substances.

No.	Component Name	The Mass Required for the Xenobiotics Utilization of 1222.7 Equivalents per Day (Equivalents → Mass), in Grams
1	Acetylcysteine (NAC)	0.266–0.327
2	Complex preparation of amino acids (leucine, lysine, phenylalanine, valine, threonine, isoleucine, methionine, histidine, tryptophan).	11.5–13.0
3	Foods with average concentrations of proteins, fats and carbohydrates (averages based on previously published data) by dry weight.	44.3–53.1
4	Foods with a high protein content (for example, 80% whey isolate).	40.1–41.9
5	Vitamin C	0.2153
6	Vitamin preparation B-100: Consisting of B_1_; B_2_; B_3_; B_6_; B_9_; B_12_, etc. (at the same time, vitamins B_3_; B_6_; B_1_; B_2)_ (given in descending order according to the corresponding molar fractions) account for approximately 71% of the total ARA).	0.879–1.404

## Data Availability

All data are presented within the article.

## References

[1] White A.P., Handler E., Smith R., Hill R., Lehman M.G. (1978). Principles of Biochemistry.

[2] Thannickal V.J. (2009). Oxygen in the Evolution of Complex Life and the Price We Pay. Am. J. Respir. Cell Mol. Biol..

[3] Taverne Y.J., Merkus D., Bogers A.J., Halliwell B., Duncker D.J., Lyons T.W. (2018). Reactive Oxygen Species: Radical Factors in the Evolution of Animal Life: A Molecular Timescale from Earth’s Earliest History to the Rise of Complex Life. BioEssays.

[4] Kehrer J.P., Klotz L.-O. (2015). Free Radicals and Related Reactive Species as Mediators of Tissue Injury and Disease: Implications for Health. Crit. Rev. Toxicol..

[5] Nohl H., Kozlov A.V., Gille L., Staniek K. (2003). Cell Respiration and Formation of Reactive Oxygen Species: Facts and Artefacts. Biochem. Soc. Trans..

[6] Harman D. (1956). Aging: A Theory Based on Free Radical and Radiation Chemistry. J. Gerontol..

[7] Grassi D., Desideri G., Ferri C. (2010). Flavonoids: Antioxidants Against Atherosclerosis. Nutrients.

[8] Chen B.T., Bechtold W.E., Barr E.B., Cheng Y.-S., Mauderly J.L., Cuddihy R.G. (1989). Comparison of Cigarette Smoke Exposure Atmospheres in Different Exposure and Puffing Modes. Inhal. Toxicol..

[9] Kutsenko S.A. (2004). Fundamentals of Toxicology: Scientific-Methodical Edition.

[10] Reinke L.A., Moore D.R., McCay P.B. (1997). Mechanisms for Metabolism of Ethanol to 1-Hydroxyethyl Radicals in Rat Liver Microsomes. Arch. Biochem. Biophys..

[11] Kowalczyk P., Sulejczak D., Kleczkowska P., Bukowska-Ośko I., Kucia M., Popiel M., Wietrak E., Kramkowski K., Wrzosek K., Kaczyńska K. (2021). Mitochondrial Oxidative Stress—A Causative Factor and Therapeutic Target in Many Diseases. IJMS.

[12] Ou B., Huang D., Hampsch-Woodill M., Flanagan J.A., Deemer E.K. (2002). Analysis of Antioxidant Activities of Common Vegetables Employing Oxygen Radical Absorbance Capacity (ORAC) and Ferric Reducing Antioxidant Power (FRAP) Assays: A Comparative Study. J. Agric. Food Chem..

[13] Lapinskii A.G., Gorbachev V.V. (2006). The Antiradical Activity of Extracts from Some Wild-Growing Plants of the Okhotsk Sea Northern Coastal Region. Pharm. Chem. J..

[14] Gorbachev V., Klokonos M., Orlovtseva O., Tefikova S., Nikitin I. (2021). Analysis of Anti-Radical Activity of Some Food Suitable Algae of the Sea of Okhotsk. E3S Web Conf..

[15] Gorbachev V.V., Klokonos M., Mutallibzoda S., Tefikova S., Orlovtseva O., Ivanova N., Posnova G., Velina D., Zavalishin I., Khayrullin M. (2022). Antiradical Potential of Food Products as a Comprehensive Measure of Their Quality. Foods.

[16] Gorbachev V., Nikitina M., Velina D., Mutallibzoda S., Nosov V., Korneva G., Terekhova A., Artemova E., Khashir B., Sokolov I. (2022). Artificial Neural Networks for Predicting Food Antiradical Potential. Appl. Sci..

[17] Jariene E., Lasinskas M., Danilcenko H., Vaitkeviciene N., Slepetiene A., Najman K., Hallmann E. (2020). Polyphenols, Antioxidant Activity and Volatile Compounds in Fermented Leaves of Medicinal Plant Rosebay Willowherb (*Chamerion angustifolium* (L.) Holub). Plants.

[18] Herdy A.H., Caixeta A. (2016). Brazilian Cardiorespiratory Fitness Classification Based on Maximum Oxygen Consumption. Arq. Bras. De Cardiol..

[19] Martinchik A.N., Laikam K.E., Kozyreva N.A., Keshabyants E.E., Mikhailov N.A., Baturin A.K., Smirnova E.A. (2021). The Prevalence of Obesity in Various Socio-Demographic Groups of the Population of Russia. Probl. Nutr..

[20] Cífková R., Bruthans J., Wohlfahrt P., Krajčoviechová A., Šulc P., Jozífová M., Eremiášová L., Pudil J., Linhart A., Widimský J. (2020). 30-Year Trends in Major Cardiovascular Risk Factors in the Czech Population, Czech MONICA and Czech Post-MONICA, 1985–2016/17. PLoS ONE.

[21] Kamadjeu R.M., Edwards R., Atanga J.S., Kiawi E.C., Unwin N., Mbanya J.-C. (2006). Anthropometry Measures and Prevalence of Obesity in the Urban Adult Population of Cameroon: An Update from the Cameroon Burden of Diabetes Baseline Survey. BMC Public Health.

[22] López-Sobaler A.M., Aparicio A., Aranceta-Bartrina J., Gil Á., González-Gross M., Serra-Majem L., Varela-Moreiras G., Ortega R.M. (2016). Overweight and General and Abdominal Obesity in a Representative Sample of Spanish Adults: Findings from the ANIBES Study. BioMed Res. Int..

[23] Maksimović M.Ž., Gudelj Rakić J.M., Vlajinac H.D., Vasiljević N.D., Nikić M.I., Marinković J.M. (2016). Comparison of Different Anthropometric Measures in the Adult Population in Serbia as Indicators of Obesity: Data from the National Health Survey 2013. Public Health Nutr..

[24] Walpole S.C., Prieto-Merino D., Edwards P., Cleland J., Stevens G., Roberts I. (2012). The Weight of Nations: An Estimation of Adult Human Biomass. BMC Public Health.

[25] Omar A.M. (2002). Determination of the relationship between the free combustion of cigarettes and the number of puffs. News High. Educ. Inst. Food Technol..

[26] (2022). Report 2021—Pesticide Residues in Food—Joint FAO/WHO Meeting on Pesticide Residues.

[27] FAO, Weltgesundheitsorganisation (2015). Pesticide Residues in Food 2014: Joint FAO WHO Meeting on Pesticide Residues.

[28] (2017). Pesticide Residues in Food–2016: Toxicological Evaluations. Proceedings of the Joint Meeting of the FAO Panel of Experts on Pesticide Residues in Food and the Environment and the WHO Core Assessment Group on Pesticide Residues.

[29] World Health Organization (2021). Food and Agriculture Organization of the United Nations Pesticide Residues in Food: 2019: Toxicological Evaluations. Proceedings of the Joint Meeting of the FAO Panel of Experts on Pesticide Residues in Food and the Environment and the WHO Core Assessment Group on Pesticide Residues.

[30] Saldan I.P., Shved O.I., Balandovich B.A., Nagornyak A.S., Mazko O.N., Makarova O.G., Filipova S.P., Szhukova O.V., Potseluev N.Y. (2018). Assessment of risks caused by Impacts exerted on a human body by nitrates contained in food Products. Health Risk Anal..

[31] Hord N.G., Tang Y., Bryan N.S. (2009). Food Sources of Nitrates and Nitrites: The Physiologic Context for Potential Health Benefits. Am. J. Clin. Nutr..

[32] Jonvik K.L., Nyakayiru J., van Dijk J.-W., Wardenaar F.C., van Loon L.J.C., Verdijk L.B. (2017). Habitual Dietary Nitrate Intake in Highly Trained Athletes. Int. J. Sport Nutr. Exerc. Metab..

[33] Salehzadeh H., Maleki A., Rezaee R., Shahmoradi B., Ponnet K. (2020). The Nitrate Content of Fresh and Cooked Vegetables and Their Health-Related Risks. PLoS ONE.

[34] Houzé P., Baud F.J., Mouy R., Bismuth C., Bourdon R., Scherrmann J.M. (1990). Toxicokinetics of Paraquat in Humans. Hum. Exp. Toxicol..

[35] Kervégant M., Merigot L., Glaizal M., Schmitt C., Tichadou L., de Haro L. (2013). Paraquat Poisonings in France during the European Ban: Experience of the Poison Control Center in Marseille. J. Med. Toxicol..

[36] Yeh S.T.-Y., Guo H.-R., Su Y.-S., Lin H.-J., Hou C.-C., Chen H.-M., Chang M.-C., Wang Y.-J. (2006). Protective Effects of N-Acetylcysteine Treatment Post Acute Paraquat Intoxication in Rats and in Human Lung Epithelial Cells. Toxicology.

[37] Glavind J. (1963). Antioxidants in animal tissue. Acta. Chem. Scand..

[38] Montville J.B., Ahuja J.K.C., Martin C.L., Heendeniya K.Y., Omolewa-Tomobi G., Steinfeldt L.C., Anand J., Adler M.E., LaComb R.P., Moshfegh A. (2013). USDA Food and Nutrient Database for Dietary Studies (FNDDS), 5.0. Procedia Food Sci..

[39] Skurikhin I.M., Tutelyan V.A. (2002). Chemical Composition of Russian Food Products: A Reference Book.

[40] Popova A.Y., Tutelyan V.A., Nikityuk D.B. (2021). Norms of Physiological Requirements in Energy and Nutrients of Various Groups of the Population of the Russian Federation. Probl. Nutr..

[41] Lyons J., Rauh-Pfeiffer A., Yu Y.M., Lu X.-M., Zurakowski D., Tompkins R.G., Ajami A.M., Young V.R., Castillo L. (2000). Blood Glutathione Synthesis Rates in Healthy Adults Receiving a Sulfur Amino Acid-Free Diet. Proc. Natl. Acad. Sci. USA.

[42] (2013). Dietary Protein Quality Evaluation in Human Nutrition: Report of an FAO Expert Consultation, 31 March–2 April, 2011, Auckland, New Zealand.

[43] Blachier F., Blais A., Elango R., Saito K., Shimomura Y., Kadowaki M., Matsumoto H. (2021). Tolerable Amounts of Amino Acids for Human Supplementation: Summary and Lessons from Published Peer-Reviewed Studies. Amino Acids.

[44] Wang X., Ouyang Y.Y., Liu J., Zhao G. (2014). Flavonoid Intake and Risk of CVD: A Systematic Review and Meta-Analysis of Prospective Cohort Studies. Br. J. Nutr..

[45] Panche A.N., Diwan A.D., Chandra S.R. (2016). Flavonoids: An Overview. J. Nutr. Sci..

[46] Kiss A.K., Bazylko A., Filipek A., Granica S., Jaszewska E., Kiarszys U., Kośmider A., Piwowarski J. (2011). Oenothein B’s Contribution to the Anti-Inflammatory and Antioxidant Activity of *Epilobium* Sp.. Phytomedicine.

[47] Naidu K.A. (2003). Vitamin C in Human Health and Disease Is Still a Mystery? An Overview. Nutr. J..

